# Challenges in the Transition of Patients with Rare Diseases from Pediatric to Adult Care: Toward an Individualized, Person-Centered Model

**DOI:** 10.3390/jpm16100525

**Published:** 2026-10-11

**Authors:** Tatiana Pire-García, Giorgina Salgueiro-Origlia, Sergio Carrasco-Molina, Lucía Ordieres-Ortega

**Affiliations:** 1 Internal Medicine Department, Hospital General Universitario Gregorio Marañón, 28007 Madrid, Spain; 2 Instituto de Investigación Sanitaria Gregorio Marañón, 28009 Madrid, Spain; 3 School of Medicine, Universidad Complutense de Madrid, 28040 Madrid, Spain; 4 Rare Diseases Working Group, Sociedad Española de Medicina Interna, 28016 Madrid, Spain; 5 Internal Medicine Department, Hospital Universitario La Paz, 28046 Madrid, Spain; 6 Instituto de Investigación Sanitaria Hospital La Paz (IdiPAZ), 28046 Madrid, Spain

**Keywords:** rare diseases, transition to adult care, pediatric-to-adult transition, continuity of care, multidisciplinary coordination, transition readiness

## Abstract

Rare diseases (RDs) collectively affect millions of people in the European Union, and advances in early diagnosis, neonatal screening, and treatment mean that increasing numbers of patients with childhood-onset RDs now reach adulthood, often with chronic sequelae and complex, long-term care needs. Transition from pediatric to adult care is therefore a critical juncture, yet it remains, in most healthcare systems, an insufficiently structured in many healthcare systems. We performed a targeted narrative review that examines the concept of biomedical literature and relevant institutional sources, prioritizing rare-disease-specific evidence while identifying evidence extrapolated from other chronic childhood-onset conditions. We examine the distinction between transition and administrative transfer; the barriers involving healthcare systems, professionals, patients, and families; and the heterogeneous evidence regarding transition outcomes. We propose a literature-informed conceptual framework centered on early identification, assessment of readiness and clinical and psychosocial complexity, named coordination, structured handover, supported decision-making, and active post-transfer follow-up. These elements support a personalized approach in which timing, intensity, professional involvement, and patient and caregiver support are adapted to individual needs rather than chronological age or diagnosis alone. European Reference Networks, patient organizations, and national support frameworks may facilitate implementation. Structured, individualized transition pathways may preserve continuity of care, treatment adherence, and patient experience, although effects on healthcare utilization and clinical outcomes require prospective evaluation.

## 1. Introduction

Rare diseases (RDs) comprise a heterogeneous group of disorders. Despite their low individual prevalence (defined in the European Union as conditions affecting fewer than five people per 10,000 inhabitants), they collectively represent a public health challenge of global scale [1]. More than 7000 distinct rare or low-prevalence clinical entities are thought to exist [2], affecting an estimated 30 million people in the European Union [3].

Historically, many of these diseases were associated with high morbidity and mortality in early life. However, advances in early diagnosis, the implementation of neonatal screening programs, and the development of targeted and supportive therapies have markedly improved prognosis [4,5]. As a result, a growing number of patients now reach adulthood [6], often carrying chronic sequelae, functional limitations, and long-term needs for highly specialized care [7].

Although some RDs are first recognized in adulthood [8], the present review focuses specifically on childhood-onset conditions requiring transfer from pediatric to adult services. The broader scarcity of adult expertise in many RDs remains relevant because it can limit the availability of receiving teams and complicate continuity after transfer [9,10,11].

Despite this progress, the transition between pediatric and adult care remains, in many settings, an insufficiently structured process with meaningful clinical and organizational consequences. Loss to follow-up and difficulties adapting to adult services have been reported in rare-disease cohorts and across other chronic childhood-onset conditions, although the magnitude and downstream clinical consequences vary substantially by disease and healthcare system [12,13,14]. These shortcomings have prompted several scientific societies and international bodies to recommend structured transition processes [15].

The heterogeneity of rare diseases also makes transition particularly relevant from the perspective of personalized medicine. A uniform pathway based primarily on chronological age is unlikely to accommodate the wide variation in disease complexity, functional and cognitive status, communication needs, treatment burden, family support, and availability of specialized adult care. Transition planning therefore represents an opportunity to extend the principles of personalized care beyond diagnosis and treatment selection to the organization and delivery of longitudinal healthcare.

This review aims to critically summarize the main challenges of transition in patients with RDs, distinguish rare-disease-specific evidence from evidence extrapolated from other chronic conditions, and propose practical, literature-informed elements for organizing an individualized transition process consistent with the principles of personalized and person-centered care within different healthcare settings.

## 2. Methods

A targeted narrative literature search was undertaken using PubMed/MEDLINE for publications available up to June 2026. Search concepts combined terms related to rare disease, chronic childhood-onset disease, adolescent or young adult, transition or transfer to adult care, transition readiness, care coordination, patient or family experience, and supported decision-making. Reference lists of relevant reviews, position papers, guidelines, and disease-specific transition studies were also screened.

Priority was given to systematic reviews, consensus or position documents, studies specifically addressing RDs, and studies reporting patient- or family-centered outcomes. Because direct evidence is limited for many individual RDs, evidence from more common chronic childhood-onset conditions was retained when it addressed transferable organizational or behavioral aspects of transition. Such evidence is identified in the text as indirect and was not assumed to be generalizable to the heterogeneous RD population. The literature was synthesized narratively; no formal risk-of-bias assessment, meta-analysis, consensus process, or prospective validation of the proposed framework was undertaken.

The framework was developed iteratively from the narrative synthesis and the authors’ clinical–organizational experience. It was further informed by the authors’ experience developing and implementing local transition protocols within their respective hospitals, particularly for patients with complex and multisystem rare diseases. The framework has not yet undergone formal prospective validation or consensus development and should therefore be regarded as a literature-informed conceptual framework requiring further evaluation.

## 3. The Concept of Transition

Care transition can be defined as the dynamic, planned, and multifactorial process encompassing the set of actions designed to help adolescents and young adults with chronic conditions move from a child-centered healthcare system to one oriented towards adults [16]. It is a deliberate, progressive process aimed at ensuring continuous care that is responsive to the patient’s needs throughout life [17], integrating not only clinical aspects but also the psychosocial, educational, and vocational dimensions specific to this stage [1]. It is important to distinguish transition from transfer, understood as the administrative act in which responsibility for care passes from the pediatric to the adult team. A well-conducted transition involves a process of preparation and support that extends well beyond that single moment [18].

The goals of a structured transition program include increasing disease knowledge, strengthening self-management skills where feasible, and supporting the highest achievable level of participation in care [19]. These goals should not be equated with complete independence. For some young people with RDs, particularly those with intellectual disability, communication difficulties, or severe functional impairment, successful transition may depend on sustained caregiver involvement and supported decision-making. Evidence from chronic-condition programs suggests improvements in satisfaction, perceived preparedness, and selected process outcomes, but effects vary across populations and interventions [20,21].

Clinically, transition seeks to preserve treatment adherence and continuity of follow-up. Structured programs have been associated with improvements in some health and utilization outcomes in selected conditions, but evidence for reductions in complications, hospital admissions, and unnecessary emergency department use, mortality, or costs is heterogeneous and is not established uniformly across RDs [12,15,22]. At an organizational level, transition tests the health system’s capacity to respond to the complexity of RDs, which are characterized by multisystem disease, dispersed expertise, and the need for effective multidisciplinary coordination and genuine continuity across care levels [23]. The process should also address needs specific to this life stage, including reproductive health, education and employment, mental health, social participation, and prevention of age-related comorbidities [24].

## 4. Stages of the Care Transition Process

Care transition should not be understood as a single event tied to a specific age, but rather as an evolving process accompanying adolescent development into adulthood. There is no universally appropriate age at which transition should begin, or transfer should occur. Timing should be individualized according to clinical stability, developmental stage, communication and cognitive abilities, functional needs, readiness, family context, and the organizational rules of the healthcare system [25,26,27].

To help structure the process, several international frameworks organize transition into distinct phases in which responsibilities are progressively redistributed among the pediatric and adult teams, the young person, and caregivers [28]. Age ranges can be useful organizational prompts, but should be interpreted as indicative rather than prescriptive.

### 4.1. Pre-Transfer Phase

The pre-transfer phase generally begins in early adolescence and continues until transfer. Its purpose is structured, progressive preparation of the young person, family, and receiving services rather than preparation of the patient alone.

During early adolescence, the goal is to begin building health literacy and familiarity with the transition process. Where developmentally appropriate, adolescents should progressively learn the name of their condition, treatments, warning symptoms, and how to communicate their needs [29]. In parallel, the pediatric team should introduce transition to the patient and caregivers and develop an individualized transition plan defining care needs, the professionals involved, responsibilities and anticipated barriers. The plan should be reviewed and updated periodically [6]. Opportunities for direct communication and confidentiality should be introduced when appropriate, while recognizing that some patients will continue to require caregiver-supported communication [19].

As transfer approaches, preparation should intensify [19]. The emphasis is on the progressive development of autonomy. Patients should gain a deeper understanding of their disease and treatments, as well as of the consequences of poor adherence or loss to follow-up. At this stage, it is essential to systematically assess transition readiness and to encourage participation in decision-making [22].

### 4.2. Transfer Phase

Transition readiness should be assessed repeatedly rather than treated as a pass/fail threshold [30].

Transfer is the administrative movement of care from pediatric to adult services and often occurs around the age of legal adulthood, although the permitted timing varies between healthcare systems [31]. It should not be equated with complete independence in healthcare management. Some young adults will manage appointments, request and renew medication, and communicate independently; others will do so with graded support from family members, caregivers, legal representatives, or professionals. Clinical readiness and self-management skills are distinct from legal decision-making capacity. The precise timing and support plan should therefore be agreed explicitly among the young person, caregivers where appropriate, and both care teams [12,32,33].

During this phase, the pediatric team should update the readiness and complexity assessment, identify unresolved risks, and ensure a structured handover containing diagnoses, current treatments, previous complications, emergency plans, pending investigations, psychosocial and communication needs, relevant legal arrangements, and named contacts. The adult team, in turn, should review the information before the first appointment and, where feasible, participate in joint or overlapping consultations. These measures may facilitate continuity, reduce duplication, and help establish trust in the receiving service [34].

When organizationally possible, transfer should be avoided during periods of marked clinical instability, major treatment change, or acute psychosocial crisis [35]. However, postponement is not always possible because pediatric age limits and service eligibility vary. If transfer must occur despite incomplete readiness or instability, the response should be increased support, explicit contingency planning, and rapid communication between teams rather than indefinite retention in pediatric care. In highly complex patients, the intensity and duration of overlap should be individualized; chronological age alone should not determine the level of support [34].

### 4.3. Post-Transfer Phase

The post-transfer phase begins with the first adult-care contact and should continue until continuity is demonstrably established rather than for a fixed duration alone [31]. Early follow-up should confirm attendance, treatment access and adherence, understanding of the adult-care plan, and the persistence of appropriate caregiver or social support. Services should have a mechanism for rapidly identifying missed appointments and initiating proactive outreach when loss to follow-up is suspected.

Transition can be considered complete when the young person is engaged with the appropriate adult team, the care plan and responsibilities are understood by the relevant participants, essential information has been transferred, and there are no unresolved handover gaps requiring pediatric ownership. Table 1 summarizes the main objectives and key interventions across phases.

## 5. Barriers to Adequate Transition

Despite growing recognition of its importance, care transition continues to face numerous obstacles that compromise its safety and quality. These barriers, consistently described in the literature, cut across countries and healthcare settings. They can be grouped into three interrelated levels: the healthcare system, professionals, and patients/families; although in practice they often overlap [36,37].

### 5.1. Health System Barriers

Fragmentation of care and disparities in resources between pediatric and adult services are the main obstacles [38]. While pediatric hospitals typically have integrated multidisciplinary teams, including specialist nurses, social workers, and psychologists, the adult system is often more fragmented and less oriented towards holistic support, which can hinder patient adaptation [39]. In addition, poor integration between the information systems of different centers can impede the appropriate transfer of complex clinical histories [40]. Organizational measures such as dedicated young-adult clinics, telephone support, or proactive outreach to patients who miss appointments can improve adherence and reduce loss to follow-up at this stage [15].

### 5.2. Barriers Related to Professionals

Limited knowledge and experience in RDs among adult-care professionals is a significant constraint [9], which can generate clinical uncertainty and complicate decision-making [10]. Pediatric teams, for their part, may find it difficult to disengage from patients with whom they have built long-standing relationships, which can hinder continuity between the two levels of care [41]. In addition, training in transition is often limited, and a clearly defined coordinating role is frequently absent [42].

### 5.3. Barriers Related to Patients and Families

These barriers are closely tied to the life stage at which transition occurs. Adolescence may combine limited disease knowledge, variable health literacy, emerging self-management skills, and competing educational or social priorities [13,14]. In a family-centered pediatric model, caregivers often hold substantial expertise and responsibility. The challenge is therefore not simply to reduce parental involvement, but to recalibrate responsibilities according to the young person’s abilities, preferences, safety, and legal context. Overprotection may hinder skill development in some patients, whereas premature withdrawal of caregiver support may be harmful in others [43,44].

Likewise, leaving a familiar pediatric team, entering an unfamiliar adult environment, and having to re-establish trust can generate distress for both young people and families [45,46]. Patients with RDs may additionally encounter adult clinicians with limited disease-specific expertise and fragmented multispecialty care [11]. Joint visits, advance introduction to the adult team, peer support, patient organizations, and clear explanations of how adult services work may facilitate adaptation [11,41,46]. Patient-reported experience measures may also help services identify transition problems that are not captured by clinical outcomes alone [25].

### 5.4. Consequences of Inadequate Transition

Suboptimal transition is consistently associated with process failures such as loss to follow-up, fragmented information transfer, and reduced treatment adherence. Associations with hospital admission, emergency department use, complications, and other clinical outcomes have also been reported, but the evidence is heterogeneous and often derives from individual diseases or from more common chronic childhood-onset conditions rather than from RDs as a whole [35,47].

Accordingly, increased morbidity, mortality, and healthcare costs should be regarded as potential consequences rather than uniform or fully demonstrated effects across the RD population. Reviews of healthcare utilization around transition have reported mixed findings, reinforcing the need to distinguish plausible benefits of structured transition from outcomes demonstrated in particular conditions or health systems [22,37].

Table 2 summarizes the main problems identified and the key strategies for addressing them in clinical practice.

## 6. A Literature-Informed Conceptual Framework for Transition in RDs

The framework proposed here is a literature-informed conceptual framework rather than a validated model. It synthesizes components repeatedly identified in published transition frameworks, RDs studies, and position papers, while adapting their delivery to challenges that are particularly relevant in RDs, including marked clinical and functional heterogeneity, variable need for caregiver support, and limited or geographically dispersed disease-specific adult expertise. Its purpose is therefore to provide an adaptable pathway for clinical implementation and future evaluation, not to prescribe a single organizational structure [11,31,48,49,50].

**Figure 1:**
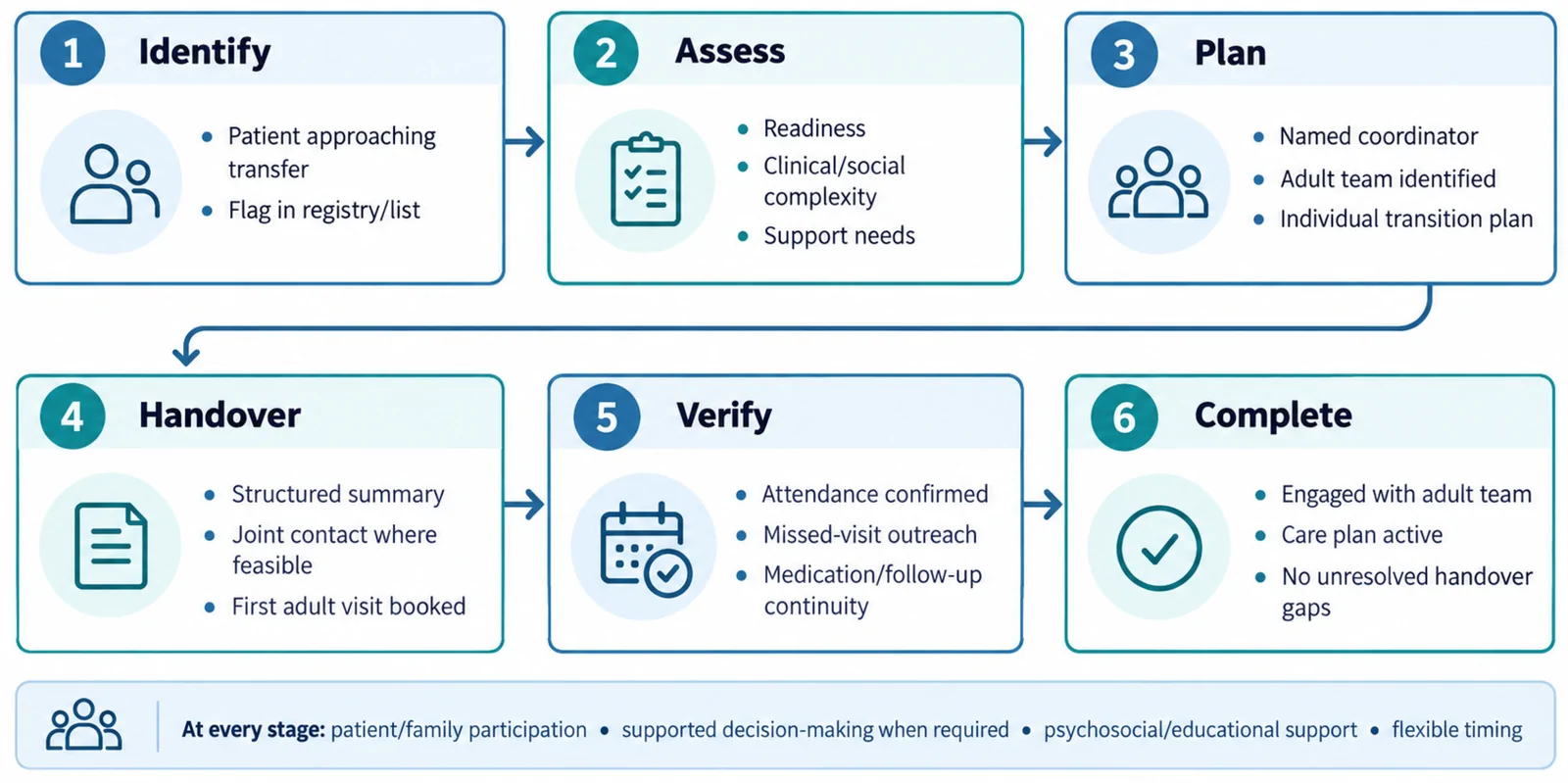
Literature-informed operational pathway for transition from pediatric to adult care in rare diseases. Timing and professional roles should be adapted to the patient, disease, and healthcare system.

Given the heterogeneity of RDs and healthcare systems, the framework prioritizes functions that should be achieved, such as identification, assessment, coordination, handover, follow-up, and confirmation of continuity, while allowing the professionals assigned to those functions to vary. Figure 1 summarizes the proposed pathway.

From a personalized-medicine perspective, the purpose of this stratification is not to create fixed categories of patients but to match the transition pathway to the individual. The timing of transfer, intensity of coordination, duration of pediatric–adult overlap, educational approach, degree of caregiver involvement, and composition of the receiving team may therefore differ substantially between patients with the same diagnosis. In this sense, personalized transition care complements disease-specific treatment by adapting healthcare delivery to the patient’s clinical, functional, psychosocial, and contextual characteristics.

### 6.1. Identification and Stratification

Early identification of patients approaching transition is the first operational step. Pediatric services should use a reproducible mechanism, such as an age-triggered list, disease registry, or periodic multidisciplinary review, to flag patients in advance of transfer [37]. Readiness should then be assessed using structured tools where appropriate. The Transition Readiness Assessment Questionnaire (TRAQ) is a patient-reported measure of transition-related skills and should not be interpreted as an objective measure of autonomy or legal capacity [51]. Its usefulness may be limited in patients with cognitive impairment, communication difficulties, low health literacy, severe disability, or substantial caregiver dependence; complementary clinician and caregiver assessment is therefore required.

In parallel, services should assess clinical and social complexity. Multisystem disease, technology dependence, cognitive or communication impairment, polypharmacy, frequent acute care, prior non-adherence, mental-health needs, social vulnerability, or geographical distance from expertise may justify more intensive coordination and longer overlap [26]. Stratification should determine the intensity of support, not eligibility for transition.

### 6.2. Care Coordination

Each patient should have a clearly identified coordinating function and a documented transition plan. The coordinator should confirm the receiving adult service, maintain a timeline, ensure completion of the handover summary, identify unresolved clinical or social issues, and verify that the first adult appointment has been arranged. The professional performing this function may be a specialist nurse, pediatrician, adult physician, social worker, primary care professional, or multidisciplinary team depending on the disease and healthcare system [23,52]. Coordination should therefore be defined by responsibilities rather than by a single universally applicable profession.

Joint consultations or overlapping pediatric–adult contact can help establish trust and clarify responsibilities. Where these are not feasible, direct clinician-to-clinician communication, virtual case discussion, and a structured handover document are alternatives [22]. The receiving team should review the history before transfer, and the patient and caregivers should receive an accessible copy of the essential care plan, medication list, emergency information, and contact pathway.

### 6.3. The Transition Coordinator

A transition coordinator can substantially facilitate navigation and continuity, but the coordinating function need not be assigned to a nurse in every setting. Specialist nurses are particularly well positioned where available because they can integrate education, care navigation, communication, and follow-up; however, physicians, social workers, primary care professionals, or multidisciplinary transition teams may undertake the same function according to local resources [53].

When a nurse case manager performs this role, responsibilities may include transition education, assessment of self-management skills, identification of psychosocial barriers, communication with patient organizations and other services, and proactive follow-up after transfer [53,54].

### 6.4. Active Involvement, Supported Decision-Making, and the Family Role

Transition should promote the highest achievable level of patient participation in decisions and disease management rather than impose independence as a universal endpoint [19]. Education should be tailored to developmental stage, health literacy, communication abilities, and functional needs. Skills may include understanding the condition and treatment, recognizing urgent problems, communicating preferences, managing medication, and navigating appointments; which of these are performed independently should vary by patient [55,56].

Clinical readiness, autonomy, and legal decision-making capacity are related but distinct concepts. Reaching the age of legal adulthood does not by itself establish that a patient can manage all aspects of care independently, nor does dependence on caregivers eliminate the patient’s right to participate. Where capacity is limited, supported decision-making should be used whenever possible, with information adapted to the person’s communication needs and preferences. Formal representation or other legal mechanisms should be considered only according to applicable national law and individual circumstances [39,46]. Families and caregivers should receive explicit guidance on how their role is expected to evolve rather than being asked simply to withdraw from care (Table 3).

### 6.5. Psychosocial Dimensions and Support Resources

The framework should explicitly incorporate psychosocial support and continuity of non-medical services such as rehabilitation, speech and communication therapy, mental-health care, social work, and educational or vocational support. Transition planning should address education, employment, sexuality and reproductive health, independent or supported living, transport, financial or social benefits, and legal decision-making where relevant [6].

Schools, universities, and vocational services may be important transition partners because healthcare transition occurs simultaneously with educational and occupational transitions. With the young person’s consent and according to local rules, coordination may be needed around attendance, treatment during school hours, emergency plans, accessibility, examinations and reasonable adjustments. Patient organizations and peer-support programs can provide disease-specific information, experiential knowledge, and practical support for families entering adult services [57].

### 6.6. European and National Facilitators and Legal Context

At the European level, the European Reference Networks (ERNs) link specialized centers for rare, low-prevalence, and complex diseases and support cross-border expert collaboration, clinical guidance, education, registries, and virtual case discussion. Several ERN and inter-ERN activities specifically address transition from pediatric to adult care [58]. Patients do not directly self-refer to an ERN; access generally occurs through healthcare providers within national arrangements. Orphanet provides searchable information on disease-specific patient organizations and alliances, which may help families identify peer and advocacy support [57].

National facilitators differ substantially. In Spain, the National Health System Rare Diseases Strategy provides a framework for coordinated and person-centered RD care and was most recently evaluated for implementation across autonomous communities [59]. Legal protections relevant to transition are generally disability- or needs-based rather than specific to an RD diagnosis [60].

### 6.7. Healthcare Organization

Training healthcare professionals is essential to improving the quality of transition. Adult clinicians may have limited exposure to childhood-onset RDs, while pediatric clinicians may be unfamiliar with adult-system pathways; targeted education and access to expert networks can reduce this knowledge gap [27,58]. Primary care, specialist adult services, reference centers, and local hospitals should have defined roles according to disease complexity and local organization.

Primary care professionals can provide longitudinal points of reference, participating in clinical follow-up, detection of comorbidities, and social and healthcare coordination. Internists, meanwhile, play a strategic role for patients with multisystem involvement or without a natural single-organ receiving specialty, but they should be presented as one possible organizational solution rather than the default coordinator in all systems. The same principle applies to the Section 7.

Transition in RDs from pediatric to adult care is a complex process that requires structured planning to ensure continuity of care, treatment adherence, and clinical stability.

Developing organizational strategies tailored to the complexity of these diseases is a healthcare priority. Communication, continuity of care, and clearly defined points of reference are key elements in facilitating patient adaptation.

Flexible organizational solutions such as telemedicine, shared-care arrangements, and hub-and-spoke networks may reduce geographical barriers to expertise [19,58]. After transfer, the coordinating team should confirm attendance at the first adult appointment and define a response to missed visits. Transition should only be closed administratively once adult-care engagement, medication access, follow-up responsibilities, and any required caregiver or legal support are confirmed.

## 7. Discussion

This review highlights a mismatch between broad agreement on the principles of good transition and the limited comparative evidence demonstrating the effectiveness of specific organizational models. RD-specific studies consistently identify fragmentation, scarcity of adult expertise, anxiety about leaving familiar pediatric teams, and the need for coordination and preparation [11,13,14,42,46,50]. However, much of the evidence on utilization, adherence, and clinical outcomes comes from individual diseases, medically complex populations, or more prevalent chronic conditions [20,22,47,61,62]. These findings are informative but should not be assumed to have identical effects across all RDs [11,13,14,42].

The strongest and most transferable elements of current evidence are therefore process-oriented: early preparation, explicit coordination, assessment of readiness and support needs, a structured handover, involvement of the receiving team before or at transfer, and active confirmation of continuity afterwards [22,25,26,27,28,31]. By contrast, evidence that transition programs reduce hospitalization, emergency care, mortality, or total costs is less consistent and is strongly influenced by the disease, intervention design, follow-up duration, and healthcare system [22,61,62]. The proposed framework consequently treats these outcomes as hypotheses for evaluation rather than guaranteed benefits.

An important implication of these findings is that personalized medicine in RDs should not be restricted to genomic diagnosis or targeted therapy. For patients with lifelong disorders, personalization also concerns how care is organized across the life course. The framework proposed here operationalizes this principle by adapting transition timing, intensity of support, coordination mechanisms, caregiver participation, and professional involvement to the patient’s clinical complexity, developmental abilities, communication needs, psychosocial context, and local availability of expertise. Two patients with the same rare disease may therefore require markedly different transition pathways. This organizational dimension of personalized medicine may be particularly relevant in RDs, where phenotypic heterogeneity, multimorbidity, disability, and uneven distribution of expertise frequently make standardized age-based transfer pathways inadequate.

Implementation also requires flexibility. The professionals available to coordinate transition, the age at which pediatric eligibility ends, access to specialized adult services, primary care capacity, and social or educational protections differ among countries and regions. Regardless of how care is organized, the pathway requires sufficient capacity to maintain clear responsibility across the handover, identify disruptions in follow-up or treatment, and obtain appropriate adult specialist input when needed. A framework based on required functions may therefore be more portable than one based on fixed professions or ages. European Reference Networks and patient organizations can facilitate expertise and information sharing, but they do not replace the need for accountable local pathways and named clinical responsibility [57,58]. A further implication is that transition quality should not be measured solely by patient independence. For some people with RDs, especially those with cognitive, communication, or severe functional limitations, the appropriate outcome is supported participation with safe continuity of caregiver involvement. Future studies should incorporate patient- and caregiver-reported experience, loss to follow-up, timeliness of the first adult appointment, treatment continuity, and completion of key handover processes, alongside disease-specific clinical outcomes. Accordingly, a personalized definition of successful transition should reflect the highest achievable level of safe participation for each individual rather than a uniform expectation of complete autonomy.

This review has several limitations. Its narrative design is susceptible to selection bias and does not provide the reproducibility or formal risk-of-bias assessment of a systematic review. The evidence base is heterogeneous, spans different diseases and healthcare systems, and frequently relies on extrapolation from chronic conditions that are not rare. The conceptual framework was derived from literature synthesis and clinical–organizational reasoning without formal stakeholder consensus, Delphi methodology, or prospective validation. Its feasibility, resource requirements, and impact should therefore be tested in different RD populations and health systems before broad implementation.

Transition from pediatric to adult care in RDs is a longitudinal process requiring preparation of the patient, caregivers, pediatric team, and receiving adult services. Its organization should be individualized according to clinical complexity, developmental and communication needs, available support, and healthcare-system constraints.

The literature supports a set of core functions: early identification, repeated assessment of readiness and complexity, named coordination, structured handover, supported participation in decision-making, and active verification of continuity after transfer. Within a personalized-medicine framework, these functions should be adapted to the individual rather than delivered through a fixed age- or diagnosis-based pathway. The age, professionals, intensity of support, and organizational structure used to deliver them should therefore remain flexible. Extending personalized care from disease-specific diagnosis and treatment to the organization of transition may help preserve continuity across the life course for people living with rare diseases.

## Figures and Tables

**Table 1:** Main objectives and interventions at each phase of transition.

Phase	Indicative Timing	Main Objectives	Key Interventions
Pre-transfer	Early adolescence until transfer (individualized)	Introduce transition; build disease knowledge. Development of autonomy and participation; identify support needs; prepare patient, caregivers, and receiving team.	Individual transition plan; repeated readiness and complexity assessment; supported participation in consultations; education tailored to cognitive, communication, and functional needs.
Transfer	Usually around legal adulthood; depends on health-system rules and individual circumstances	Safe administrative transfer with continuity of clinical responsibility and information.	Named adult service; structured handover; first appointment booked; joint/overlapping contact when feasible; contingency plan if transfer occurs during instability.
Post-transfer	From first adult-care visit until continuity is established	Integration into adult care; prevention of loss to follow-up; clarification of ongoing support and decision-making arrangements.	Confirm attendance and treatment access; proactive outreach after missed visits; reassess support needs; document completion criteria.

**Table 2:** Barriers that may arise during the transition process, impact, and potential solutions.

Problem	Impact	Key Strategies
Dispersed expertise across centers	Difficulty maintaining continuity and follow-up	Networked model: coordination between the expert center and the local hospital
Lack of coordination between levels of care	Loss of information and fragmented care	Structured transition plan and standardized clinical reports
Absence of an adult-care point of contact	Unintegrated follow-up in complex patients	Designation of a coordinating function and named adult-care contact
Limited self-management skills or need for supported decision-making	Risk of poor adherence or loss to follow-up if support is mismatched to needs	Tailored education, readiness assessment, caregiver support, and supported decision-making
Limited involvement of primary care	Weak longitudinal continuity	Early involvement of the primary care physician
Territorial/logistical barriers	Limited access to specialized centers	Hybrid models with telemedicine and shared follow-up

**Table 3:** Practical actions for patients and caregivers during transition.

Stage	Patient/Caregiver Actions	Purpose
Early preparation	Ask when transition will begin; maintain an updated medication and problem list; identify who currently coordinates care; discuss the young person’s preferred level of involvement.	Make transition visible early and establish shared expectations.
Readiness and planning	Practice age- and ability-appropriate tasks; identify communication, cognitive, mobility, educational, social, and legal support needs; ask which adult team will receive care.	Match responsibilities and support to individual needs.
Before transfer	Request an accessible clinical summary, emergency plan, contact details, prescription plan, and confirmed first adult appointment; meet the adult team where feasible.	Reduce information loss and uncertainty.
At transfer	Clarify who is responsible for urgent problems, prescriptions, test results, and pending referrals during the handover period.	Avoid gaps in clinical responsibility.
After transfer	Attend the first adult appointment; confirm that medication and follow-up continue; contact the service promptly after missed appointments or unresolved access problems; give feedback on the transition experience.	Detect loss to follow-up and unresolved handover problems early.

## Data Availability

No new data were created or analyzed in this study. Data sharing is not applicable to this article.

## Abbreviations

The following abbreviations are used in this manuscript:

Abbreviation	Definition
RD	Rare disease
TRAQ	Transition Readiness Assessment Questionnaire
